# Surgically Treated Carpal Tunnel Syndrome and Ulnar Nerve Entrapment at the Elbow in Different Occupations and their Effect on Surgical Outcome

**DOI:** 10.1097/JOM.0000000000002539

**Published:** 2022-05-04

**Authors:** Filippa Linde, Mattias Rydberg, Malin Zimmerman

**Affiliations:** From the Department of Orthopaedics, Helsingborg Hospital, Helsingborg, Sweden (Filippa Linde, Dr Malin Zimmerman); Department of Translational Medicine – Hand Surgery, Lund University, Malmö, Sweden (Filippa Linde, Dr Mattias Rydberg, Dr Malin Zimmerman); Department of Hand Surgery, Skåne University Hospital, Malmö, Sweden (Dr Mattias Rydberg).; From the Department of Orthopaedics, Helsingborg Hospital, Helsingborg, Sweden (Filippa Linde, Dr Malin Zimmerman); Department of Translational Medicine – Hand Surgery, Lund University, Malmö, Sweden (Filippa Linde, Dr Mattias Rydberg, Dr Malin Zimmerman); Department of Hand Surgery, Skåne University Hospital, Malmö, Sweden (Dr Mattias Rydberg).

**Keywords:** carpal tunnel syndrome, occupations, risk factors, surveys and questionnaires, ulnar nerve compression syndromes

## Abstract

We investigated whether certain occupations were over-represented among surgically treated carpal tunnel syndrome and ulnar entrapment at the elbow, and if manual occupation affected surgical outcome.

**Methods:**

We included 9030 patients operated for CTS and 1269 for UNE registered in the Swedish National Quality Register for Hand Surgery (HAKIR) 2010–2016. Occupational data was retrieved from Statistics Sweden. Outcome was assessed using the QuickDASH questionnaire.

**Results:**

In patients operated for CTS, there were more assistant nurses, attendants/care providers/personal assistants, nannies/student assistants, carpenters/bricklayers/construction workers, cleaners, nurses, and vehicle mechanics than in the general population. In the UNE population, assistant nurses and attendants/care providers/personal assistants were over-represented. Manual workers with CTS scored the preoperative QuickDASH higher than non-manual workers.

**Conclusions:**

Manual workers are overrepresented among surgically treated CTS and UNE. Manual workers with CTS have more symptoms preoperatively than non-manual workers.

## Abstract 2

This study provides an overview on occupational demographics of a population with surgically treated carpal tunnel syndrome (CTS) and ulnar nerve entrapment at the elbow (UNE). Manual workers with CTS had more symptoms preoperatively than non-manual workers, which might be useful information for treating physicians when counseling patients.

## INTRODUCTION

Occupational factors might play a role in the development of peripheral nerve entrapments in the upper extremity. The most common peripheral nerve entrapment, carpal tunnel syndrome (CTS), affects approximately 3% of the general population.^1^ Risk factors include female sex, diabetes and rheumatoid arthritis.^1,2^

There are also occupational factors, where repetitive manual work tasks involving extension and flexion of the wrist, vibrations of the arm and hand, and forceful hand grip are established risk factors for CTS.^3–5^ Office workers with intensive computer use might be at greater risk of developing CTS,^6^ as well as dentists.^7^

The gold standard of surgical treatment of CTS is open carpal tunnel release (OCTR).^8^ The prognosis after OCTR depends on multiple factors, including type of occupation, where exposure to heavy lifting, bending of the wrists, and repetitive movements extend the time before return to work.^9^ It is unclear how the patient’s occupation affects self-reported symptoms and disability before and after OCTR.

The second most common nerve entrapment in the upper extremity is ulnar nerve entrapment (UNE) at elbow level.^10^ Smoking, heavy work, and low education level are risk factors for UNE.^11^ One occupational risk factor is “holding a tool in position.” There is also a greater risk of developing UNE when another musculoskeletal disorder, primarily medial epicondylitis or CTS, is already present.^12^ Moderate UNE can be treated with an orthosis, and more severe symptoms may be treated surgically.^13^ The prognosis after UNE surgery has been insufficiently studied and there are few articles to be found concerning the effect of the patient’s occupation on the prognosis. In one systematic review from 2017, it seems that heavy manual workers are at risk of more severe UNE and worse prognosis following surgery.^14^

The distribution of different occupations in surgically treated CTS and UNE in Sweden is not known. Hence, the aim of this study was firstly to investigate whether people with certain occupations are over-represented in surgically treated CTS or UNE. Secondly, this study aimed to investigate whether preoperative symptoms and the subjective outcome of surgery are affected by the patient’s occupation.

## METHODS

This is a retrospective study of patients with CTS (ICD-10 diagnosis code G650, surgical KKÅ 97 code ACC51) or UNE at the elbow (ICD-10 diagnosis code G652, surgical KKÅ 97 codes ACC43, ACC53, or NCK19) that underwent surgery from 2010–2016. Patients were included from the Swedish National Quality Register for Hand Surgery (HAKIR; www.hakir.se). Information about patient occupations was obtained from Statistics Sweden (SCB). The patients’ unique ten-digit personal identification numbers were used for register linkage.

### Study Design

HAKIR is a Swedish national quality register managed by the Swedish Hand Surgery Association. All seven Swedish university hospitals are part of the register as well as two private healthcare providers. Inclusion in the register is optional and patients can resign from participation whenever they wish. Data are collected on four different occasions: before and during surgery, and at three and 12 months postoperatively. The patient fills out a questionnaire (patient-reported outcome measure, PROM) before surgery and three and 12 months postoperatively.^15^ The PROM used is the QuickDASH questionnaire.

QuickDASH is the short version of the DASH Outcome Measure. DASH stands for disabilities of arm, shoulder and hand. The shortened questionnaire uses 11 items to measure symptoms and physical function in the upper limb.^16^ The QuickDASH items are scored between 0 and 5. A total score is then calculated, ranging between 0 and 100, where 0 corresponds to no symptoms and 100 to worst possible discomfort.^17^ The Swedish translation of the QuickDASH questionnaire was used.^18^

SCB uses a syntax called Swedish Standard Classification of Occupations (SSYK) to classify individual occupations. SSYK originates from the International Standard Classification of Occupations 2008 (ISCO-08).^19^ In the present study, a person was classified as an employee if they had an income above the established limit or, if self-employed, their income derived from active business operations. The established limit (level of income) was determined at the time by a specific model with information obtained from the Swedish workforce survey (AKU) and control data from SCB; this applies both to employees and the self-employed. According to AKU a person should have worked at least one hour a week in the month of November, or be temporarily absent from work, in order to be classified as having an occupation.^20,21^ Non-employees and people with missing data concerning occupation, could be students, senior citizens, unemployed, people on sick leave, or employees in another country.^20,21^

We classified occupations that included work tasks involving the hands as manual occupations since we considered this a more modern classification than grouping by blue-collar or white-collar work.^22^

### Statistics

When comparing the outcome after surgery, each treated hand was considered a separate statistical entity. In the calculations regarding occupation and when comparing proportions to those in the general population, patients who had bilateral surgery during the study period were only included once. Data are presented as the number of people in the populations with each specific occupation. The proportions of each occupation against the whole population were calculated using the chi-square test. The non-parametric Mann – Whitney U test was used to identify differences in QuickDASH scores between patients with manual and non-manual occupations, as well as to investigate potential sex differences. A *P* value <0.05 was considered statistically significant. The data were analyzed using IBM SPSS Statistics version 27 (SPSS Inc., Chicago, IL) and Microsoft Excel version 16.48 (Microsoft Corporations, Redmond, WA). Proportions were compared using MedCalc Software Ltd version 20.009.^23^

## RESULTS

In total, we identified 10,746 hands treated surgically for CTS during the study period. Of these, 1717 patients were operated on bilaterally for CTS during the study period. Hence, 9030 patients operated on for CTS were included in the study.

During the study period, there 1346 arms with ulnar nerve entrapment registered in HAKIR. Of these, 77 patients were operated on bilaterally, resulting in 1269 included patients.

In both populations, manual occupations were more common among men (Tables 1 and 2). More patients with manual occupations had UNE together with another upper extremity disorder, such as trigger finger or CTS, compared to non-manual workers (*P* = 0.027, data not shown).

**TABLE 1:** Ten Most Common Occupations in 2015 in 3822 Patients with Surgically Treated Carpal Tunnel Syndrome

Occupation	Employees (*n* = 3822)	Men (*n* = 1202)	Women (*n* = 2620)
Age at surgery, years	50 [40 – 57]	51 [43 – 59]	49 [38 – 56]
Manual occupation	2178 (69)	738 (61)	1440 (55)
Assistant nurse (SSYK 532)	352 (9.2)	5 (0.4)	347 (13.2)
Primary school teacher, after-school teacher, and pre-school teacher (SSYK 234)	192 (5.0)	9 (0.7)	183 (7.0)
Attendant, care provider, and personal assistant (SSYK 534)	188 (4.9)	19 (1.6)	169 (6.5)
Clerical worker and secretary (SSYK 411)	181 (4.7)	12 (1.0)	169 (6.5)
Nanny, student assistant etc (SSYK 531)	155 (4.1)	3 (0.2)	153 (5.8)
Shop worker (SSYK 522)	159 (4.2)	27 (2.2)	132 (5.0)
Carpenter, bricklayer, and construction worker (SSYK 711)	136 (3.6)	132 (11.0)	4 (0.2)
Cleaners, home-help staff etc (SSYK 911)	116 (3.0)	6 (0.5)	110 (4.2)
Nurse (SSYK 222)	117 (3.1)	5 (0.4)	112 (4.3)
Light vehicle technician and repairer etc (SSYK 723)	83 (2.2)	80 (6.7)	3 (0.1)

In 2015, 5075 of the original patient population (*n* = 9030) had an occupation. Of these, data on type of occupation were missing in 1253 patients. Age presented as median [interquartile range; IQR]. All other data presented as *n* (%).

**TABLE 2:** Ten Most Common Occupations in 2015 in 715 Patients with Surgically Treated Ulnar Entrapment at the Elbow

Occupation	Employees (*n* = 494)	Men (*n* = 259)	Women (*n* = 235)
Age at surgery, years	48 [39 – 55]	49 [41 – 56]	46 [38 – 55]
Manual occupation	271 (55)	153 (59)	118 (50)
Assistant nurse (SSYK 532)	40 (8.1)	7 (2.7)	33 (14.0)
Attendant, care provider, and personal assistant (SSYK 534)	28 (5.7)	5 (1.9)	23 (9.8)
Clerical worker and secretary (SSYK 411)	18 (3.6)	4 (1.5)	14 (6.0)
Primary school teacher, after-school teacher, and pre-school teacher (SSYK 234)	18 (3.6)	3 (1.2)	15 (6.4)
Nanny, student’s assistant etc. (SSYK 531)	18 (3.6)	3 (1.2)	15 (6.4)
Warehouse staff and transport manager (SSYK 432)	15 (3.0)	13 (5.0)	2 (0.9)
Cleaner, home-help staff etc. (SSYK 911)	14 (2.8)	3 (1.2)	11 (4.7)
Shop worker (SSYK 522)	16 (3.2)	4 (1.5)	12 (5.1)
Truck and bus driver (SSYK 833)	15 (3.0)	12 (4.6)	3 (1.3)
Engineer and technician (SSYK 311)	14 (2.8)	11 (4.2)	3 (1.3)

In 2015, 715 of the original patient population (*n* = 1269) had an occupation. Of these, data on type of occupation were missing in 221. Age presented as median [interquartile range; IQR]. All other data presented as *n* (%).

### Occupations

Of the 9030 included patients operated for CTS, 5075 patients were occupied/employed in 2015. Data on type of occupation was missing in 1253/5075 (25%). The most common occupation was assistant nurse with 352/3822 employees (9% of all patients with CTS), followed by primary school teacher/after-school teacher and pre-school teacher (192/3822; 5%) and attendant/care provider/personal assistant (188/3822; 5%; Table 1). Three of the ten most common occupations for those with CTS were not found among the top ten occupations in Sweden for the same year (Table 1 compared to Table 3). These occupations were cleaner/home-help staff, etc, nurse, and light vehicle technician/repairer. The following occupations were more common among the CTS population than in the general population: assistant nurse (9.2% vs 4.6%; *P* < 0.0001), attendant/care provider/personal assistant (4.9% vs 3.8%; *P* = 0.0003), nanny/student assistant etc. (4.1 vs 2.6; *P* < 0.0001), carpenter/bricklayer/construction worker (3.6% vs 2.4%; *P* < 0.0001), cleaner/home-help staff etc. (3.0% vs 1.9%; *P* < 0.0001), nurse (3.1% vs 2.0%; *P* < 0.0001) and light vehicle technician/repairer etc. (2.2% vs 1.5%; *P* = 0.0002).

**TABLE 3:** Ten Most Common Occupations in the Swedish Population Aged 18–64 Years, in 2015

Occupation	Occupation (*n* = 4,051,459)	Men (*n* = 2,038,424)	Women (*n* = 2,013,035)
Shop worker (SSYK 522)	221,619 (5.2)	83,688 (4.1)	137,931 (6.9)
Assistant nurse (SSYK 532)	184,435 (4.5)	15,245 (0.7)	169,190 (8.4)
Primary school teacher, after-school teacher and pre-school teacher (SSYK 234)	183,762 (4.3)	31,452 (1.5)	152,310 (7.6)
Clerical worker and secretary (SSYK 411)	172,515 (4.0)	30,900 (1.5)	141,615 (7.0)
Attendant, care provider, and personal assistant (SSYK 534)	153,471 (3.6)	40,442 (2.0)	113,028 (5.6)
Insurance adviser, business salesperson, and buyer (SSYK 332)	135,499 (3.2)	89,385 (4.4)	46,114 (2.3)
Nanny, student’s assistant etc (SSYK 531)	106,755 (2.5)	17,911 (0.9)	88,844 (4.4)
System architect, software developer, test leader etc (SSYK 251)	104,584 (2.4)	81,629 (4.0)	22,955 (1.1)
Carpenter, bricklayer and construction worker (SSYK 711)	98,568 (2.3)	96,413 (4.7)	2,155 (0.1)
Engineer and technician (SSYK 311)	93,927 (2.2)	76,961 (3.8)	16,966 (0.8)

Data presented as *n* (%). Occupation unknown for 231,558 employees who were excluded from the above calculations.

Shop workers were less common in the CTS population than in the general population (4.2% vs 5.5%; *P* < 0.0001).

In 2015, there were no data regarding occupation for 740/1269 (58%) of the patients surgically treated for UNE. In this population as well, the most common occupation was assistant nurse with 40/715 (8%) employees, followed by attendant/care provider/personal assistant (28/715, 6%), and the third most common occupation was clerical worker/secretary (18/715, 4%; Table 2). Distribution differed in three occupations between the UNE population and the general Swedish population in 2015 (Table 2 compared to Table 3). These occupations were warehouse staff/transport manager, cleaner/home-help staff etc., and truck and bus driver. The following occupations were more common in the UNE population than in the general population: assistant nurse (8.1% vs 4.6%; *P* = 0.0002) and attendant/care provider/personal assistant (5.7% vs 3.8%; *P* = 0.029), while shop workers were less common (3.2% vs 5.5%; *P* = 0.029).

The SSYK3 register of occupations in Sweden for 2015 contained 4,277,634 people; for 237,953 of these occupations was unknown because of missing data. The three most common occupations in the Swedish population in 2015 were shop worker 221,619/4,051,459 (5.2%), assistant nurse 184,435/4,051,459 (4.3%) and primary school teacher/after-school teacher/pre-school teacher, 183,762/ 4,051,459 (4.3%; Table 3).

### Outcome for Patients With a Manual Occupation Compared to Patients With a Non-manual Occupation

In the population with CTS, preoperative QuickDASH scores were higher in manual than in non-manual workers (Table 4). In women with a manual occupation, there was a larger improvement in QuickDASH score from preoperatively to three months postoperatively compared to women with a non-manual occupation (*P* = 0.012). However, women with non-manual occupations had a larger improvement from three to twelve months postoperatively (*P* = 0.005). In general, women scored higher (worse symptoms) in the QuickDASH than men regardless of type of occupation.

**TABLE 4:** QuickDASH Results Preoperatively, at Three and 12 Months Postoperatively After Surgical Treatment of Carpal Tunnel Syndrome and Ulnar Nerve Entrapment at the Elbow, Divided into Manual or Non-manual Occupation

	CTS						UNE					
	Men, *n* = 3596			Women, *n* = 7150			Men, *n* = 697			Women, *n* = 649		
	Non-manual	Manual	*P* Value	Non-manual	Manual	*P* Value	Non-manual	Manual	*P* Value	Non-manual	Manual	*P* Value
Total preoperative	34	41	**0.029**	48	52	**0.028**	32	41	0.056	59	57	0.949
QuickDASH score	[23–55] (*n* = 215)	[27–57] (*n* = 319)		[34–64] (*n* = 544)	[36–66] (*n* = 671)		[16–51] (*n* = 42)	[25–59] (*n* = 64)		[36–69] (*n* = 46)	[38–72] (*n* = 49)	
Total QuickDASH score	17	18	0.724	20	18	0.446	20	25	0.253	20	32	0.863
three months postoperative	[9–30] (*n* = 146)	[8–32] (*n* = 205)		[11–36] (*n* = 405)	[9–36] (*n* = 437)		[7–36] (*n* = 24)	[11–41] (*n* = 38)		[16–45] (*n* = 30)	[9–52] (*n* = 47)	
Total QuickDASH score	11	9	0.524	11	14	0.185	14	23	0.236	39	29	0.392
12 months postoperative	[2–31] (*n* = 93)	[0–28] (*n* = 117)		[2–27] (*n* = 298)	[5–30] (*n* = 308)		[5–30] (*n* = 27)	[9–45] (*n* = 20)		[18–55] (*n* = 40)	[11–53] (*n* = 30)	
Change in QuickDASH	16	20	0.194	20	25	0.012	6	13	0.235	22	30	0.946
score 0–3 months	[5–25] (*n* = 71)	[7–33] (*n* = 81)		[9–34] (*n* = 210)	[11–41] (*n* = 213)		[2–18] (*n* = 10)	[3–40] (*n* = 16)		[12–48] (*n* = 12)	[12–43] (*n* = 24)	
Change in QuickDASH	5	5	0.386	7	5	0.005	6	4,5	1.000	2	1	0.927
score 3–12 months	[_3–14] (*n* = 57)	[0–14] (*n* = 75)		[0–16] (*n* = 175)	[_3–11] (*n* = 182)		[_2–13] (*n* = 13)	[_4–18] (*n* = 12)		[_14–7] (*n* = 19)	[_8–14] (*n* = 18)	
Change in QuickDASH	18	22	0.192	27	27	0.916	9	5	0.834	9	1	0.082
score 0–12 months	[5–31] (*n* = 44)	[11–50] (*n* = 46)		[14–41] (*n* = 152)	[14–43] (*n* = 136)		[_12–16] (*n* = 9)	[_4–18] (*n* = 10)		[_11–27] (*n* = 11)	[_8–14] (*n* = 13)	

Evaluated QuickDASH-score, where 0 represents no problem and 100 worst possible problems. The score in QuickDASH for each date and the change in evaluated QuickDASH score preoperatively -3 months postoperatively, 3–12 months postoperatively, and preoperatively-12 months postoperatively. Data are median [IQR]. Each operated hand was considered a separate statistical entity. Number of responders for each occasion is presented below each value (*n*).

Statistical significance was calculated using the Mann–Whitney U test with a *P* value<0.05 considered statistically significant.

CTS, carpal tunnel syndrome; UNE, ulnar nerve entrapment at the elbow.

No significant difference in QuickDASH scores, either pre-operatively or three or twelve months postoperatively, between manual and non-manual occupations was found for surgically treated UNE.

## DISCUSSION

The first aim of this study was to investigate whether people with certain occupations are over-represented in surgically treated CTS or UNE. We found that several occupations were more common among people operated on for CTS and UNE than in the general population. There are several plausible explanations to this. It might imply that these employees are at higher risk of developing CTS and UNE severe enough to require surgical treatment. It is, however, possible that people with manual occupations are more prone to seek specialized care and accept surgical treatment for CTS and UNE than people with non-manual occupations, where CTS and UNE might interfere less with work. In non-manual occupations, there might also be more room for adjustment of work tasks as needed to relieve symptoms. For the same reasons, it is plausible that people with manual work seek help earlier than people with non-manual work. It is also possible that people with manual work tasks and non-specific activity-related pain seek care and are diagnosed with CTS and UNE, which might be incidental to the symptoms of pain. The present results are in line with a previous multicenter study from Italy where blue-collar workers, with heavy manual work tasks, had a higher risk of surgically treated CTS.^22^

The most common occupation in both populations was assistant nurse, which is a predominantly female occupation, according to SCB’s occupational register.^24^ Female sex is a well- known risk factor for developing CTS (1, 4), which might be part of the explanation as to why assistant nurses were over-represented in both populations. Assistant nurse was the second most common occupation in Sweden in 2015.^24^

In the CTS population, there were more of light vehicle technicians/repairers and carpenters/bricklayers/construction workers than in the general population. Their work tasks require forceful hand movements together with flexion and extension of the wrist, which are known risk factors for CTS,^3^ as well as forceful hand-grip work, which was shown by Jackson et al to be a risk factor in a cohort of Swedish construction workers.^25^

Truck and bus drivers were more common in the UNE population than in the general population. One risk factor for UNE is holding a tool in position,^12^ and driving requires holding the steering wheel in position. A study of Turkish taxi drivers found that leaning the elbow against the car window might cause ulnar entrapment at the elbow because of both long-term flexion and mechanical pressure.^26^

Manual occupations are over-represented in the ten most common occupations in both CTS and UNE groups. Even though the results of this study cannot establish any causative connection between manual work and surgically treated CTS, it is possible that people with manual work could be helped by proactive measures. Proactive measures could include more education about work tasks involving risk, to encourage the workers to be more cautious and the employers to improve the work environment to avoid occupational injuries. Ergonomic interventions could also constitute a preventive action, however, a meta-analysis of ergonomic interventions and office workers could find no evidence of an association between better ergonomics and lower risk of upper extremity disorders.^27^

The second aim of this study was to investigate the outcome after surgery and its association with the patient’s type of occupation.

The results of this study indicate that patients with manual occupations and CTS, from both sexes, experience more symptoms and impaired function preoperatively than patients with non-manual occupations and CTS. This may be because manual workers are more dependent on their upper extremities in their everyday work and therefore experience more disability. It is also possible that manual workers present with worse CTS than non-manual workers, if they have work tasks that aggravates the nerve compression.

This study also indicates that female manual workers experience a greater improvement in function straight after surgery whereas for female non-manual workers the improvement seems to be more long-term. One can speculate whether that is because manual workers have more symptoms preoperatively and therefore experience a bigger improvement right after surgery. However, manual work requires longer sick-leave following open carpal tunnel release (in our experience, six weeks is often advised for manual workers to allow the ligament to heal). Therefore, manual workers might need a rehabilitation regime that differs from that for non-manual workers. This is a target for further research.

There is a connection between socioeconomic deprivation and the risk of UNE, where people with a low educational level, heavy dependence on social assistance, and extensive sick leave appear to suffer from UNE to a greater extent.^28^ Long-term sick leave, regardless of reason, negatively affects the patient’s outcome after surgery.^28^ Even though we were unable to find any differences between the type of occupation and outcome after surgery for UNE, it is still of importance to search further for variables affecting the outcome after surgery.

The risk of developing UNE is higher in people who already have one musculoskeletal disorder, such as CTS.^12^ The present study confirms that there is a higher risk of developing UNE and a concomitant upper extremity disorder if the patient has a manual occupation.

One strength of this study is the use of the short version of the QuickDASH questionnaire, since it might be less of a burden for the patient to fill in fewer questions making it more appealing to answer. Gummeson’s et al^29^ article from 2006 indicates that QuickDASH has the same reliability in measuring symptoms as the primary DASH version with 30 questions. This study only represents surgically treated carpal tunnel syndrome and ulnar nerve entrapment at the elbow and cannot automatically be applied to all clinical cases of CTS/UNE.

One limitation of the study is the low response rate in QuickDASH. A potential bias is that patients answering the surveys postoperatively may be those with persistent symptoms. Another response bias, a more specific demand bias, might be that the responders are prompted to score their symptoms as being worse because they think it will influence whether or not they will get help.

Another aspect to bear in mind is the fact that the rate of missing responses is generally higher with questionnaires than, for example, with telephone interviews.^30^ Prospective studies might consider using both questionnaires and telephone interviews to follow up the outcome after surgery in order to avoid loss of answers. We also had no data on symptom duration and time before return to work.

A further limitation, that needs to be considered, is the number of patients with missing data regarding occupation for the selected year, especially in the UNE population. The amount of missing data might negatively influence the top ten occupations and distort the conclusions about occupations at risk. We chose to include only the patients with an occupation in 2015 even though not everyone was operated on that year. Future studies might consider examining the patient’s occupation for the same year in which they were operated on. To be able to obtain a sufficient number of patients surgically treated for CTS/UNE, we included all patients who appeared in HAKIR over six years. To confirm the present results, a future study could include all surgically treated patients from one specific year using the national patient register. However, this register only includes surgical codes and no PROMs.
